# Impaired Telomere Maintenance and Decreased Canonical WNT Signaling but Normal Ribosome Biogenesis in Induced Pluripotent Stem Cells from X-Linked Dyskeratosis Congenita Patients

**DOI:** 10.1371/journal.pone.0127414

**Published:** 2015-05-18

**Authors:** Bai-Wei Gu, Marisa Apicella, Jason Mills, Jian-Meng Fan, Dara A. Reeves, Deborah French, Gregory M. Podsakoff, Monica Bessler, Philip J. Mason

**Affiliations:** 1 Department of Pediatrics, Division of Hematology, The Children’s Hospital of Philadelphia, Philadelphia, PA, United States of America; 2 Department of Pathology and Laboratory Medicine, The Children’s Hospital of Philadelphia, Philadelphia, PA, United States of America; 3 Division of Hematology, Center for Cellular and Molecular Therapeutics, The Children’s Hospital of Philadelphia, Philadelphia, PA, United States of America; University of Nebraska Medical Center, UNITED STATES

## Abstract

Dyskeratosis congenita (DC) is an inherited bone marrow failure syndrome characterized by the presence of short telomeres at presentation. Mutations in ten different genes, whose products are involved in the telomere maintenance pathway, have been shown to cause DC. The X-linked form is the most common form of the disease and is caused by mutations in the gene *DKC1*, encoding the protein dyskerin. Dyskerin is required for the assembly and stability of telomerase and is also involved in ribosomal RNA (rRNA) processing where it converts specific uridines to pseudouridine. DC is thought to result from failure to maintain tissues, like blood, that are renewed by stem cell activity, but research into pathogenic mechanisms has been hampered by the difficulty of obtaining stem cells from patients. We reasoned that induced pluripotent stem (iPS) cells from X-linked DC patients may provide information about the mechanisms involved. Here we describe the production of iPS cells from DC patients with *DKC1 *mutations *Q31E*, *A353V *and *ΔL37*. In addition we constructed “corrected” lines with a copy of the wild type dyskerin cDNA expressed from the AAVS1 safe harbor locus. We show that in iPS cells with *DKC1 *mutations telomere maintenance is compromised with short telomere lengths and decreased telomerase activity. The degree to which telomere lengths are affected by expression of telomerase during reprograming, or with ectopic expression of wild type dyskerin, is variable. The recurrent mutation *A353V *shows the most severe effect on telomere maintenance. *A353V *cells but not *Q31E *or *ΔL37 *cells, are refractory to correction by expression of wild type *DKC1* cDNA. Because dyskerin is involved in both telomere maintenance and ribosome biogenesis it has been postulated that defective ribosome biogenesis and translation may contribute to the disease phenotype. Evidence from mouse and zebra fish models has supported the involvement of ribosome biogenesis but primary cells from human patients have so far not shown defects in pseudouridylation or ribosomal RNA processing. None of the mutant iPS cells presented here show decreased pseudouridine levels in rRNA or defective rRNA processing suggesting telomere maintenance defects account for most of the phenotype of X-linked DC. Finally gene expression analysis of the iPS cells shows that WNT signaling is significantly decreased in all mutant cells, raising the possibility that defective WNT signaling may contribute to disease pathogenesis.

## Introduction

Dyskeratosis congenita (DC) is an inherited bone marrow failure (BMF) syndrome characterized by the classical triad of mucocutaneous features comprising nail dystrophy, leukoplakia and abnormal skin pigmentation[1,2]. BMF is present in many patients and is the major cause of death. DC patients have an elevated risk of leukemia, solid tumors, aplastic anemia and Myelodysplastic Syndromes (MDS). So far, 10 genes have been discovered whose mutation causes DC and together they account for about 60% of patients[3]. The products of all these genes are involved in telomere maintenance and DC patients usually have very short telomeres compared to healthy controls[4,5].The most common X-linked form of DC is caused by mutations in the *DKC1* gene, encoding dyskerin[6]. Dyskerin is a highly conserved nucleolar protein that, as part of a specialized nucleolar RNP, an H/ACA snoRNP, catalyzes the pseudouridylation of specific uridine residues in newly synthesized ribosomal RNAs and spliceosomal snRNAs[7]. SnoRNPs consist of 4 proteins and an integral guide RNA, called an H/ACA snoRNA because of its conserved motifs, that localizes the template uridines by base pairing[8]. In vertebrates dyskerin, and the other 3 proteins, are also found associated with telomerase in telomerase RNP[4], which contains an integral RNA, *TERC*, that contains the template for addition of the telomere TTAGGG repeats that form the telomeric DNA[9]. The 3’ portion of *TERC*, that binds to dyskerin, resembles an H/ACA snoRNA[10]. Dyskerin is required for the assembly and stability of telomerase RNP [11,12]and therefore for maintaining the integrity of telomeres, the nucleoprotein structures that protect the ends of chromosomes from degradation and from being recognized as double strand breaks by the cell’s DNA repair system[13]. Evidence suggests that dysfunctional telomeres resulting in premature cellular senescence are the primary cause of bone marrow failure in DC[14]. However it has been suggested that impaired ribosome biogenesis and translation may contribute to the phenotype of X-linked DC[15–17]. This idea has credibility from the fact that some other BMF disorders, notably Diamond Blackfan Anemia and Shwachman Diamond Syndrome clearly result from ribosomal defects and have been called ribosomopathies[18]. Data supporting the involvement of ribosome biogenesis or translation come mainly from model organisms[16,19] while studies in patient cells have found no or little effect[20,21].

X-linked DC is highly variable in its severity, ranging from severe BMF with in utero growth retardation, cerebellar hypoplasia and immunodeficiency presenting in young children (Hoyeraal Hreidarsson syndrome, HH) to BMF and mucocutaneous problems presenting in the teens (classical DC)[5]. Most of the mutations in *DKC1* are missense mutations and they are clustered, in the 3D structure, in a region important for RNA binding though the relationship between the severity of DC and the position of the mutations in dyskerin is still unclear[22,23]. One mutation, *DKC1* ^*A353V*^ accounts for about 40 percent of X-linked DC patients and is usually *de novo* and causes a very severe clinical phenotype with BMF, very short telomere length and classical mucocutaneous features and sometimes HH[5,24]. We generated a mouse ES cell line carrying the *A353V* mutation and mutant ES cells had decreased *TERC*, accelerated telomere shortening, delayed rRNA processing and decreased levels of pseudouridine in rRNA but mice with the mutation could not be obtained[19,25]. The development of the ability to derive induced pluripotent stem cells (iPSCs) from primary human cells [26,27]gave us the opportunity to obtain human cells that express telomerase and have features of stem cells, which are the cells defective in DC.

In this study we generated iPS cells from patients’ skin fibroblast cells carrying *DKC1* mutations *Q31E*, *ΔL37* and *A353V* and found that these iPS cells showed impaired telomerase function but little, if any, lack of pseudouridine or ribosome biogenesis defect. We also show that dysfunctional telomere maintenance caused by the *A353V* mutation cannot be rescued through expression of WT dyskerin. Finally, we found that pathogenic *DKC1* mutations impair WNT/Frizzled signaling.

## Materials and Methods

### Generation and culture of iPS cells

iPSCs were generated from human fibroblast cells by expressing OCT4, SOX2, KLF4, and MYC using polycistronic lentiviral vector STEMCCA provided by G. Mostoslavsky (Boston University, Boston, MA, USA). One week after transduction, fibroblasts were transferred to plates coated with mouse embryonic fibroblasts (MEFs) and grown in iPS cell medium (ES medium, DMEM/F12, supplemented with 20% knockout serum replacement, 0.1 mM nonessential amino acids, 1 mM l-glutamine [Invitrogen, Carlsbad, CA USA], 10 ng/ml recombinant human fibroblast like growth factor–basic (Peprotech, NJ, USA.), and 0.1 mM 2-mercaptoethanol (Sigma, St Louis, MO USA). Fibroblasts containing the *DKC1* ^*ΔL37*^ mutation (GM01774) were obtained from from the Coriell Institute (GM01774, Camden, NJ, USA). The Penn-CHOP Bone Marrow Failure Syndrome (BMFS) cohort is an open prospective/retrospective cohort for the study of molecular mechanisms of BMFS, approved by the Institutional Review Boards of Children’s Hospital of Philadelphia (CHOP) and of the University of Pennsylvania (Penn). Written informed consent from all study participants or their legal guardians was obtained prior to study participation in accordance with the Declaration of Helsinki.

### Teratoma formation assay

Immune-deficient Jax-NOD.Cg-Prkdc^Scid^ Il2rg^tm1wjl^/SzJ (NSG) male and female mice at about 12 weeks of age were used as recipients for teratoma assays. Subcutaneous and intramuscular injections were performed. iPSCs were harvested and resuspended in 200μl PBS. 2.0 × 10^6^ cells were injected per site. Following development of visible tumors between 2–4 months, mice were euthanized by carbon dioxide inhalation and cervical dislocation and tumors were removed, fixed in Bouin’s solution (Sigma-Aldrich), and processed (mounting and H&E staining) by the Children's Hospital of Philadelphia Pathology core facility.

### Pulse Chase Analysis of rRNA Processing

iPS cells were preincubated for 45 min in methionine-free medium and then incubated for 30min in medium containing L-[methyl-^3^H]methionine (50 μCi/ml). The cells were then chased in nonradioactive fresh medium for various times. Total RNA was separated on 1.25% agarose formaldehyde gel and transferred onto nylon membrane. The membranes were sprayed with EN3HANCE Spray (Perkin Elmer,Waltham, MA, USA) and exposed to X-ray films at -80°C.

### Analysis of pseudouridylation in 28S and rRNA

iPS cells were cultured in phosphate-free DMEM medium for 1 h and then labeled for 3 h with [^32^P]orthophosphate (0.1 mCi/ml). Total RNA was extracted by using TRIzol Reagent (Invitrogen) and electrophoresed through a 1% agarose formaldehyde gel. The 28S and 18S rRNA were purified by electroelution, phenolchloroform extracted, ethanol precipitated, and digested with RNase T_2_ (Sigma) in 50 mM ammonium acetate, pH 4.5/0.05% SDS/1 mM EDTA at 37°C. Digested RNA (20,000 cpm each) was analyzed by two-dimensional cellulose TLC (EM Science, Gibbstown, NJ) by using isobutyric acid/NH_4_OH/H_2_O (577:38:385, by volume) in the first dimension and 2-propanol/HCl/H_2_O (70:15:15, by volume) in the second dimension. The TLC plates were exposed to X-ray films at -80°C.

### TOP-flash Luciferase Assays

A total of 5μg TOP-flash plasmid (OT-flash or OF-flash) together with pRL-SV40 plasmid (Renilla luciferase internal control) was transfected into shDKC1-HEK293T cells by using Xfect transfection kit (Clontech Laboratories, Mountain View, CA, USA). 1μM Doxycycline was added into cells to induce knock down of dyskerin protein after 24 hours of transfection. 5 μM GSK3b inhibitor (CHIR-99021) was added into cells 24 hour before harvesting the cells. Luciferase assays were performed using the Promega duel- luciferase assay system (Promega, Madison, Wisconsin, USA). Three replicate experiments were performed.

### RNA isolation

Total RNA was extracted from iPS cells and 293T cells by using TRIzol Reagent (Invitrogen, Carlsbad, CA). A rotor-stator homogenizer was used to thoroughly disrupt and homogenize tissues. RNA was aliquoted and stored at -80°C for further use.

### Northern blot analysis

10μg total RNA was electrophoresed through denaturing agarose gels and transferred onto Hybond-N membranes by using the classical high-salt buffer method. A 451bp fragment from the TERC gene was used as probe for detecting TERC expression.

### Real-time RT/PCR

Real-time RT/PCR was carried out using Power SYBR-Green PCR Master Mix (Applied Biosystems,Grand Island, NY,USA) and Superscript II MMLV transcriptase and RNase inhibitors (Invitrogen) and a 7900HT Real-time PCR system equipped with SDS software (Applied Biosystems, Grand Island, NY, USA).

### Western Blot analysis

Total protein from cells and was prepared by using RIPA lysis buffer (1×TBS, 1% NP-40, 0.5% sodium deoxycholate, 0.1% SDS, 0.004% sodium azide and 1× protease inhibitor cocktail). Protein concentration was measured by using the Bio-Rad protein assay (Bio-Rad).

### Immunofluorescence

Immunofluorescence was performed with a standard paraformaldehyde technique (fixed in PBS buffered 4% paraformaldehyde for 10 minutes, permeabilized with 0.25% Triton-PBS for 10 minutes, blocked with 10% normal goat serum for one hour). Primary antibody was used at 1/1000 in 1.5% normal goat serum for two hours. After washing with PBS, cells were incubated with a secondary goat anti-rabbit or mouse IgG conjugated with FITC or Alexa 568 at 1/1000 in 1.5% normal goat serum for 45 minutes. All blocking and incubation steps were carried out at room temperature. Finally, slides were counterstained with 4,6-diamidino-2-phenylindole (DAPI) and covered by mounting media. The cells were examined at 1000× magnification using a fluorescence microscope (Nikon, Melville, NY, USA). FITC, Alexa 568 and DAPI images were overlapped by using the Advanced software.

### Antibodies

The sources of antibodies were as follows: anti-dyskerin was as previously described, anti-NHP2 (Abcam 172481), anti-fibrillarin (Abcam 5821 and 4566), anti-NAF1(home made) anti-β-Actin was used as total protein loading control (Abcam, ab20272).

### Measurement of telomere length

iPS cells were embedded in agarose plugs by using CHEF agarose plug kit according to the manufacturer’s instructions (Bio-Rad, Hercules, CA, USA). DNA embedded in the plug was extracted, digested with Mbo I and Hinf I and electrophoresed through a 1% agarose gel for 24 hours at 6V/cm, 1-6seconds switch time using CHEF DR-III pulse-field system (Bio-Rad). 32P-^γ^-ATP labeled (CCCTAA)4 probe was used in the in-gel hybridization procedure.

### Telomerase Repeat Amplification Protocol (TRAP)

iPS cells were lysed by using the 3-[(3-cholamidopropyl)dimethylammonio]-1-propanesulfonate (CHAPS) lysis buffer. Telomerase activity was measured by TRAP assay, using the TRAP-EZE telomerase detection kit (Millipore,Billerica, Massachusetts USA), according to the manufacturer’s protocol.

### Inducible shRNA

Stable cell lines containing inducible *DKC1* shRNA was carried out by using Block-it Inducible H1 RNAi system (Invitrogene, Carlsbad, CA). pENT/H1/TO-shRNA-*DKC1* plasmid and pLenti6/TR plasmid were co- transfected into HEK-293T cells by using Xfect Transfection Reagent according to the protocol (Clontech Laboratories, Mountain View, CA, USA). Stable cells were selected by using Zeocin (400μg/ml) and Blasticidin (1 μg/ml). Inducible *DKC1* knock down was carried out by adding Doxycycline (250ng/ml) into the culture medium for 72 hours.

### Gene targeting in human iPSCs using AAVS1 zinc-finger nucleases

Zinc-finger nuclease (ZFN) cDNAs under the control of the PGK promoter were cloned into a plasmid expression vector (pPGK-ZFN-L(left) and pPGK-ZFN-R(right)). The donor construct was targeted to the AAVS1 locus using the AAVS1-SA-2A-puro-pA plasmid (Addgene) containing human *DKC1* cDNA driven by the chicken β actin promoter. Approximately 1x10^5^ iPSCs were plated onto puromycin-resistant MEF feeder cells with 10 μM Rock inhibitor (Cayman Chemical, Ann Arbor, MI, USA), then transfected using X-tremeGENE 9 (Roche, Indianapolis, IN, USA). Puromycin (0.5 μg/mL) was added 2 days later. After 2 weeks, individual clones were picked, expanded and characterized. iPSC clones were screened for heterozygous integration at the AAVS1 locus using Southern blot and PCR methods.

### Transcriptome analysis

Total RNA was isolated from cells using the RNeasy kit (Qiagen, Redwood City, CA). The level of whole genome transcripts was measured by an Affymetrix GeneChip human transcriptome array (Affymetrix, Santa Clara,CA). Data were analyzed by Partek software (Partek, Saint Louis, MO), and pathway analysis carried out using Ingenuity variant analysis (Qiagen). The original microarray repository information can be found at Gene Expression Omnibus (GEO) database with the accession number GSE66849. (http://www.ncbi.nlm.nih.gov/geo/query/acc.cgi?acc=GSE66849).

### Statistical analysis

The Student t test was performed and P values were determined using the 2-tailed t test for groups with equal variance.

## Results

### Generation of iPS cells

iPS cell lines were generated from 1. a 21-year-old male patient with a *DKC1* ^*A353V*^ mutation, very short telomeres and severe DC. 2. a 50-year-old male patient with *DKC1* ^*Q31E*^ mutation, short telomeres and mild DC (S1 Fig). 3. commercially available skin fibroblast cells carrying a DKC1^ΔL37^ mutation and 4. a 14-year-old male patient who is a compound heterozygote for *TERT*, carrying a *R537H* mutation and a *c*.*2173-2187del15insACAG* insertion/deletion (*TERT CP*). We used the STEMCCA lentiviral vector to reprogram these skin fibroblast cells to iPS cells as previously described [28,29]. Southern blot analysis was used to identify cell lines containing a single lentiviral integration site, and the reprogramming gene cassettes were subsequently removed by CRE recombinase–mediated excision [30]. All lines that were analyzed further displayed normal embryonic stem cell (ESC)-like morphology, normal karyotype, expression of endogenous pluripotency markers, and the capacity to form cells representing 3 germ layers in teratomas (S2 and S3 Figs). We used two male WT iPS cells from the Children's Hospital of Philadelphia (CHOP) iPS cell core facility to serve as control cells. There were no significant differences in terms of proliferation rate or the presence of the pluripotency markers between WT and mutant cells when kept in culture for 70 passages (data not shown).

### Impaired telomerase function in *DKC1* mutant iPS cells

**Fig 1:**
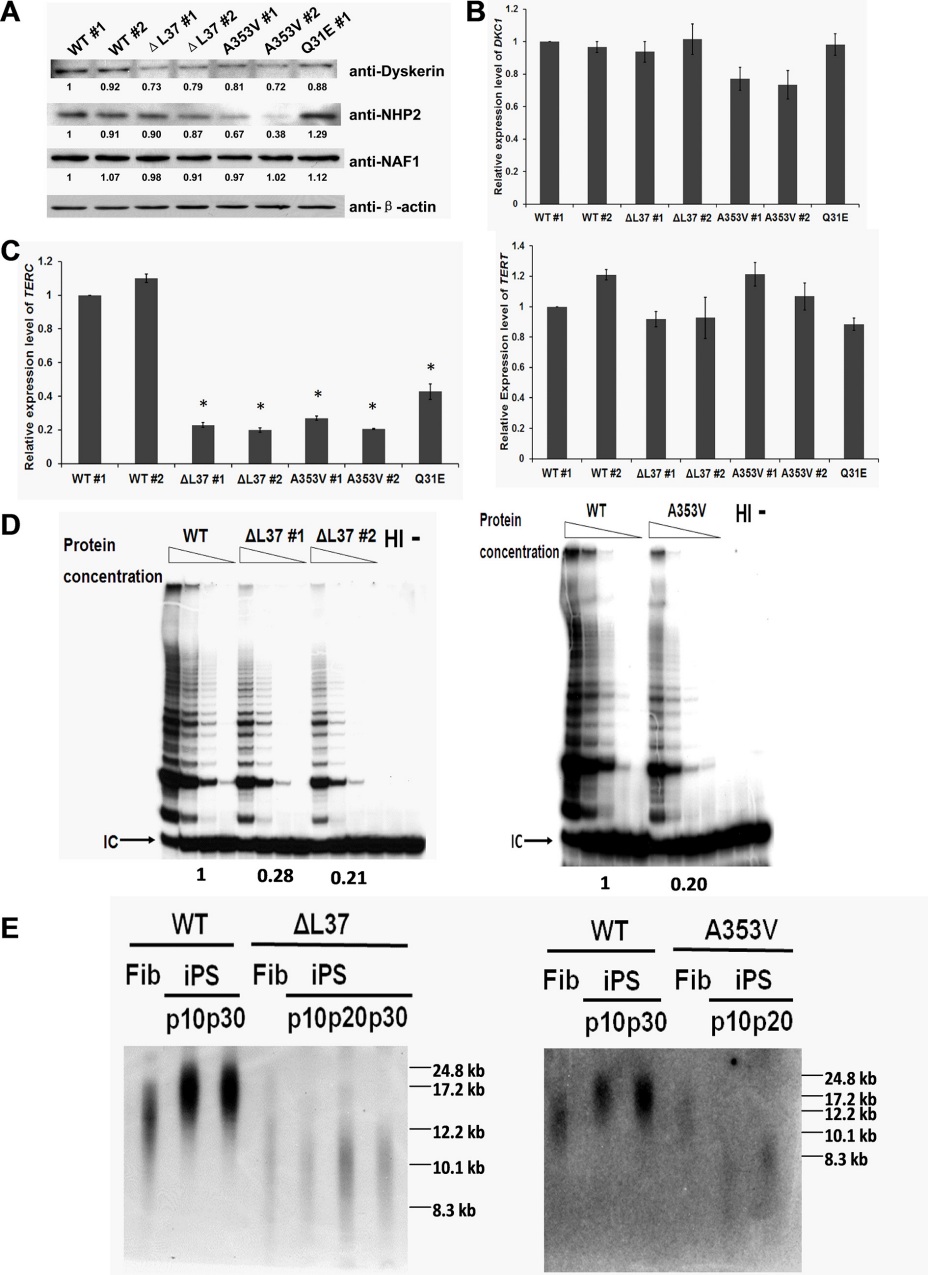
*DKC1* mutations impair telomerase function in iPS cells. A: Western blot showing levels of dyskerin, NHP2 and NAF1 proteins in the iPS cells. All mutant cells show slightly decreased level of dyskerin protein compared to WT cells. Note that *A353V* iPS cells show decreased level of NHP2 while *ΔL37* and *Q31E* cells don’t. The quantitive data, derived by densitometry, are shown. B: Real-time RT/PCR of RNA from iPS cells from DC patients and healthy controls. *ΔL37* and *Q31E* mutant *DKC1* iPS cells express same amount of *DKC1* mRNA as WT iPS cells, but *A353V* mutant iPS cells show a slightly decreased level of *DKC1* mRNA. *GAPDH* was used at loading control. The combined results of 4 independent experiments are shown, the error bars show standard deviation. C: Real-Time RT/PCR of *TERC* RNA (left) and *TERT* mRNA (right) in iPS cells. *GAPDH* was used at loading control. The combined results of 3 independent experiments are shown, the error bars show standard deviation. * p<0.01 between WT cells and different mutant cells, respectively. D: Telomerase activity assay of *ΔL37* (left) and *A353V* (right) iPS cells compared to WT cells by using TRAP assay. 2×10^6^ cells were extracted by using CHAPS lysis buffer and serial dilutions (50ng, 10ng, 2ng and 0.4ng) of each sample were assayed. IC: internal control, HI: heat inactivation. The quantitive data, derived by densitometry, are shown. E: Telomere length measurement of the iPS cells in different passages compared to those from the original fibroblast cells (Fib) by using pulse field gel electrophoresis and in-gel hybridization with telomere probe (TTAGGG)_3_.

*DKC1* mutant iPS cells showed decreased levels of dyskerin compared with WT cells with the *Q31E* mutant cells having the highest levels and *A353V* the lowest (Fig 1A). The cells showed no, or minimal (*A353V*), reduction in *DKC1* mRNA levels, suggesting that the mutant proteins are relatively unstable (Fig 1B). *A353V* mutant cells, and to some extent *ΔL37* cells showed a decrease in the levels of NHP2 but no change in NAF1 levels (Fig 1A). NAF1 is associated with the snoRNPs at very early stages of biogenesis[31] while NHP2 is present in the mature particles[32] so these levels imply that normal levels of snoRNPs are produced that are relatively unstable. Immunofluorescence staining using anti-dyskerin antibody showed that both WT and mutant dyskerin proteins are located predominantly in the nucleoli as expected (S4 Fig). We studied the expression of *TERC* RNA and *TERT* mRNA in these cells and found that all of these mutant iPS cells expressed very low levels of *TERC* RNA, only about 20–30% compared to WT iPS cells, suggesting that the mutant dyskerin proteins destabilize *TERC* RNA. As expected there was no significant change in *TERT* mRNA levels in the mutant cells Fig 1C. Next, we measured the telomerase activity of these mutant iPS cells by using the TRAP assay, a well-established *in vitro* assay for examining telomerase activity. We found that both *A353V* and *ΔL37* cells showed very low telomerase activity (about 20%) compared to WT cells (Fig 1D). Surprisingly, *Q31E* iPS cells did not show any reduction in telomerase activity compared to WT iPS cells (S5 Fig), and the telomere length of Q31E iPS cells is the same as that of the original fibroblast cells (S6 Fig). Finally, we measured telomere lengths by using Southern blot and found that the telomere length of WT iPS cells was increased compared with the fibroblasts from which the iPS cells had been reprogrammed. This is likely due to the transient increase in TERT expression that takes place during the reprograming process [26]. In *ΔL37* cells, however, the telomeres did not get elongated. Interestingly, in *A353V* iPS cells, the telomere length of *A353V* iPS was significantly shorter than the corresponding fibroblast cells, suggesting the *A353V* mutation could not maintain the telomere length during the reprograming process (Fig 1E).

### *DKC1* mutant iPS cells do not show significant defects in ribosome biogenesis

**Fig 2:**
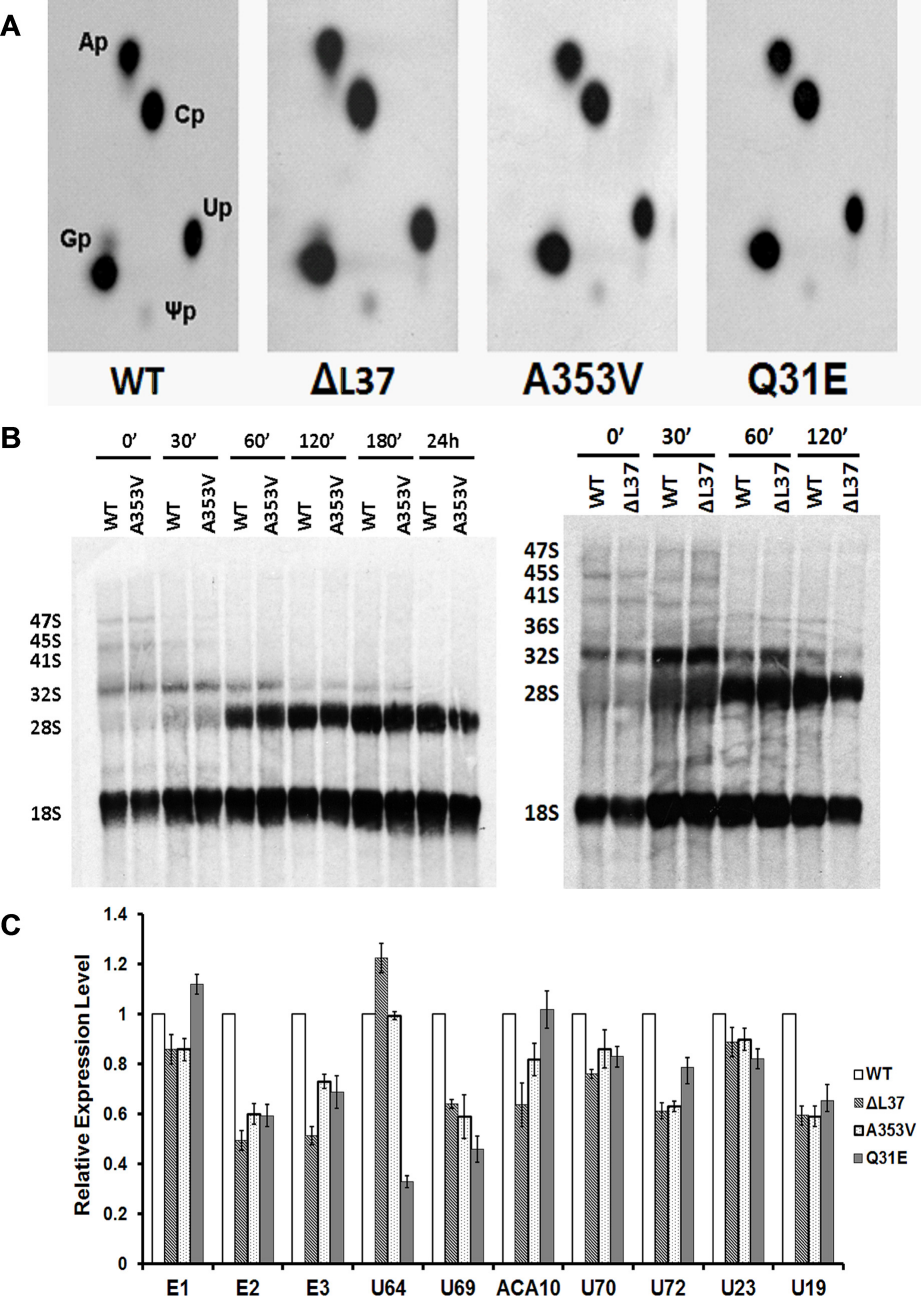
*DKC1* mutant iPS cells show no significant defects in ribosome biogenesis. A: measurement of pseudouridine in 28S rRNA from WT and mutant iPS cells. iPS cells were labeled with ^32^P-labeled orthophosphate and 28S rRNA was gel-purified. After digestion with RNase T_2_, each sample was separated by two-dimensional TLC. The positions of the labeled ribonucleotides are indicated. Ap: Adenine, Cp: Cytosine, Gp: Guanine, Up: Uridine, Ψp: Pseudouridine: B: Pulse–chase labeling experiments of rRNA isolated from *WT*, *A353V* and *ΔL37* iPS cells. Cells were labeled with L-[^3^H-methyl] methionine for 30 min and then chased in nonradioactive medium for the times shown. The RNA was separated on a 1.25% agarose gel, transferred to a nylon filter, and exposed to x-ray film. C: Real-time RT/PCR results of some H/ACA snoRNAs of *WT* and *DKC1* mutant iPS cells. Results were expressed relative to *GAPDH* RNA. The combined results of 3 independent experiments are shown, the error bars show standard deviation.

Various animal models of DC have shown clear defects in pseudouridylation of rRNA and increased accumulation of rRNA precursors[16,19], but only minimal defects have been reported from limited studies of patient cells [20,21]. We were therefore interested to assess these parameters in our mutant iPS cells. First we compared pseudouridine content by labeling cells with ^32^P-labeled phosphate, purifying 28S rRNA and analyzing the nucleotide content by thin layer chromatography. No difference in the relative amounts of uridine and pseudouridine is seen between mutant and wild type cells (Fig 2A). Next, we used a very sensitive pulse chase experiment in which cells were labelled with L-[methyl-^3^H] methionine and chased with cold methionine. Since methionine pools are low the experiment is very sensitive in detecting species with increased half lives and has been used to detect rRNA processing defects in animal models of X-linked DC [16,19,33]. *A353V* and *ΔL37* cells did not show any differences in the kinetics of rRNA processing (Fig 2B). Then, we checked the expression of some snoRNAs in these iPS cells, including some H/ACA, scaRNA and C/D snoRNA. We found that most of the H/ACA snoRNAs and scaRNAs we investigated were decreased in mutant cells while all C/D snoRNA were unchanged compared to WT iPS cells (Fig 2C and S7 Fig). In addition mobility shift under partially denaturing conditions has been shown to be a sensitive indicator of differences in pseudouridylation[34]. While rRNAs from WT and mutant iPS cells both showed the denaturation dependent shift there was no mobility difference between them (S8 Fig).

### Variable rescue of impaired telomerase function by expressing WT dyskerin

**Fig 3:**
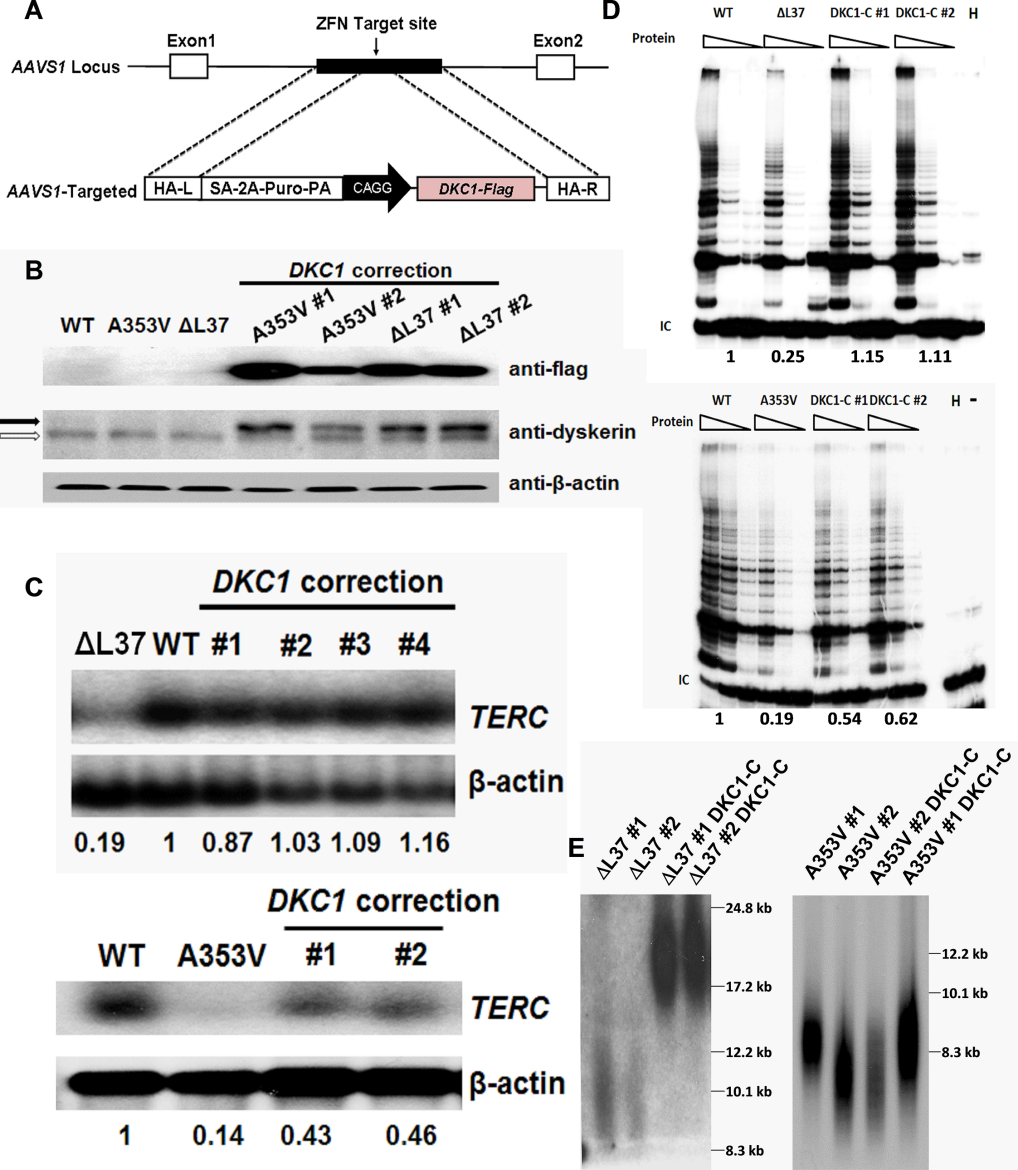
Expression of WT Dyskerin can rescue dysfunctional telomerase in *ΔL37* cells but not in *A353V* cells. A: Schematic representation of zinc-finger nuclease (ZNF)-mediated homologous recombination. The constitutively active *AAVS1* “safe harbor” locus is shown on the top line and the targeting construct is shown below. The cDNA expression cassettes driving expression of Flag-tagged WT dyskerin under the chicken actin promoter (CAGG) were inserted by zinc finger-mediated homologous recombination into intron 1 of *AAVS1*. HA, homologous arms left (L) and right (R); SA-2APuro-PA, puromycin drug resistance cassette. [30] B: Western blot showing expression of flag-tagged dyskerin protein in corrected *A353V* and *ΔL37* iPS cells by using anti-Flag antibody and anti-dyskerin antibody. Open arrow: endogenous dyskerin protein; filled arrow: Flag-tagged WT dyskerin protein. C: Northern blot result of *TERC* RNA expression levels of *DKC1* corrected iPS cells. Different corrected lines are shown as well as the uncorrected line carrying the *DKC1 ΔL37* or the *DKC1 A353V* mutation. *β-actin* was used as loading control. The quantitive data, derived by densitometry, are shown. D: The TRAP assay was performed to measure the telomerase activity after expressing the WT *DKC1* gene in *A353V* and *ΔL37* iPS cells, uncorrected and corrected by ectopic expression of WT *DKC1 (DKC1-C)*. The quantitive data, derived by densitometry, are shown E: Telomere lengths of *DKC1* corrected iPS cells were measured by using in-gel hybridization with a telomere probe (TTAGGG)_3_. *DKC1-C* indicates corrected iPS cells expressing WT *DKC1* gene.

We were interested in rescuing the short telomere phenotype for several reasons. First we wanted to know if telomere shortening in DC is reversible. Second we were interested in whether *DKC1* mutants would exert a dominant negative effect on the WT protein. This is not known because in either male or female cells only one allele is expressed per cell. Finally if we could lengthen telomeres by inserting WT dyskerin and then excise the WT dyskerin cassette we would have a cell with a *DKC1* mutation and long telomeres, in which the early stages of telomere shortening due to *DKC1* mutations could be investigated. We used a zinc finger nuclease recombination system to introduce a Flag-tagged WT *DKC1* cDNA expression vector into the “safe harbor” *AAVS1* locus [35] (Fig 3A) of *A353V* and *ΔL37* cells. We obtained some clones with a single heterozygous integration according to Southern blot analysis and studied two corrected lines for both mutations. First, we checked the protein expression level of the Flag-tagged WT dyskerin proteins in these corrected iPS cells and found that Flag-tagged WT dyskerin expression is similar in all corrected lines; The expression of Flag-tagged dyskerin is higher than endogenous mutant dyskerin protein, immunofluorescence staining by using anti-flag antibody confirmed nucleolar localization (Fig 3B and S9 Fig). Then, we looked at the *TERC* expression level after introducing the WT *DKC1* gene into *A353V* and *ΔL37* iPS cells. Northern blot analysis showed that the level of mature *TERC* RNA was fully restored in corrected *ΔL37* iPS cells but was only partially restored in corrected *A353V* cells (Fig 3C). Next, we examined the telomerase activity of these corrected iPS cells by using the TRAP assay. Consistent with the *TERC* RNA levels, corrected *ΔL37* cells showed fully rescued telomerase activity while corrected *A353V* cells only showed partially rescued telomerase activity (Fig 3D). More interestingly, when we measured the telomere length of these corrected iPS cells, we found that in *ΔL37* cells, the telomeres of corrected cells were significantly longer than the original *ΔL37* cells. However, the corrected *A353V* iPS cells could not elongate their telomeres by expressing WT dyskerin protein (Fig 3E). These results suggest a possible dominant negative effect of *DKC1* mutations which would have to be considered in any future therapeutic approaches that involve expressing WT dyskerin.

### Altered WNT/Frizzled signaling in *DKC1* mutant iPS cells

**Fig 4:**
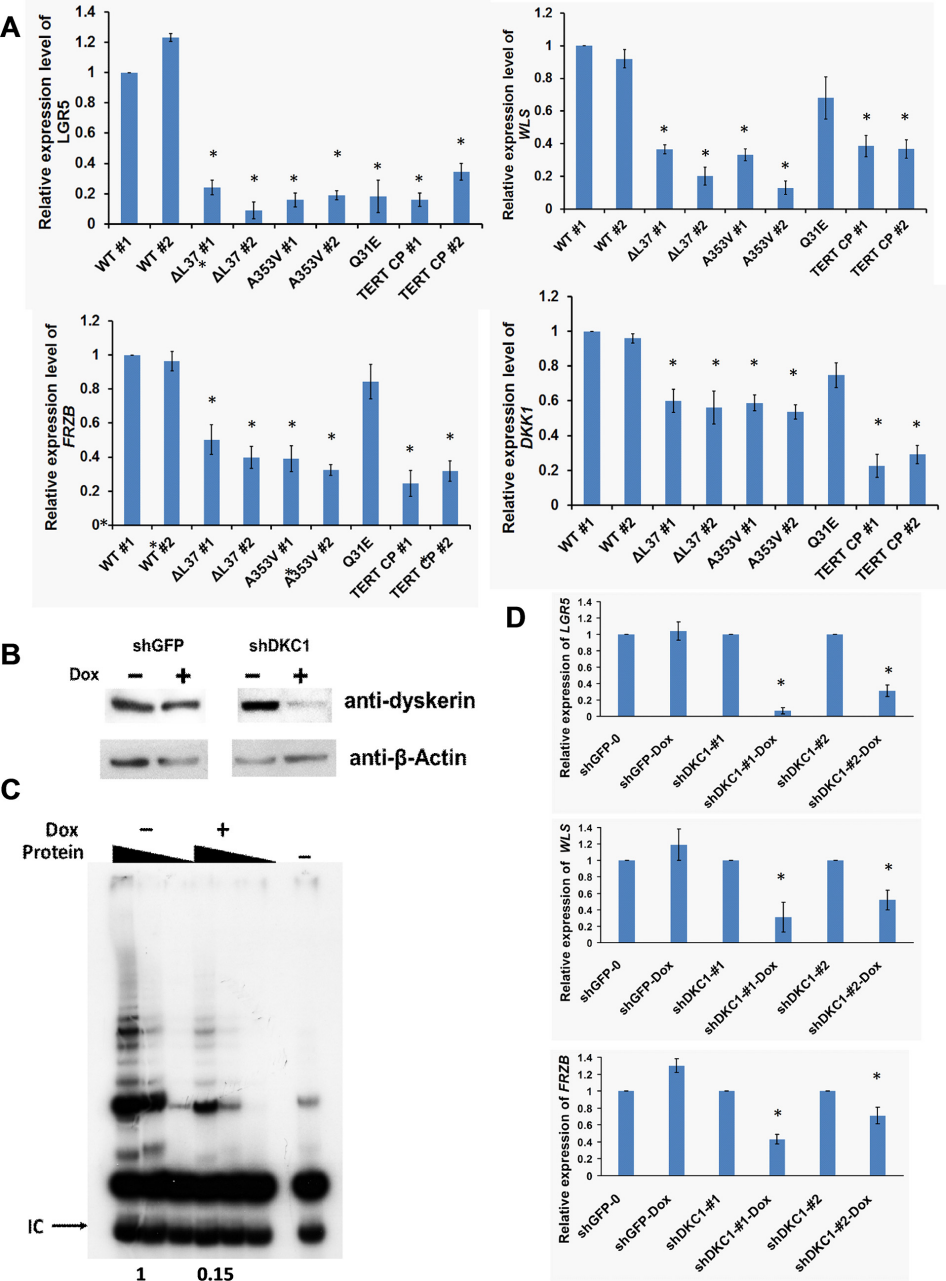
WNT/Frizzled signaling was impaired in *DKC1* mutant iPS cells. A: Validation of the results from microarray. *LGR5*, *DKK1*, *WLS* and *FRZB* mRNA expression were measured by real-time RT/PCR. 3 independent experiments were performed and the error bars show standard deviation. TERT CP: iPS cells with *TERT* ^*R537H/2173-2187del15insACAG*^ compound homozygotes mutation * p<0.01 between WT iPS cells and different mutant iPS cells, respectively. B: Western blot result of dyskerin protein in HEK293T cells showing doxycycline inducible *DKC1* shRNA can reduce the dyskerin level to about 10–20%. shGFP was used as a control. C: Telomerase activity of HEK293T cells after knocking down of dyskerin protein from B was measured by TRAP assay. The quantitive data, derived by densitometry, are shown. D; Knock down of dyskerin protein can directly decrease the expression of *LGR5*, *WLS* and *FRZB* genes. Two different *DKC1* shRNA lines were used in this experiment. The combined results of 3 independent experiments are shown, the error bars show standard deviation. * p<0.01.

**Fig 5:**
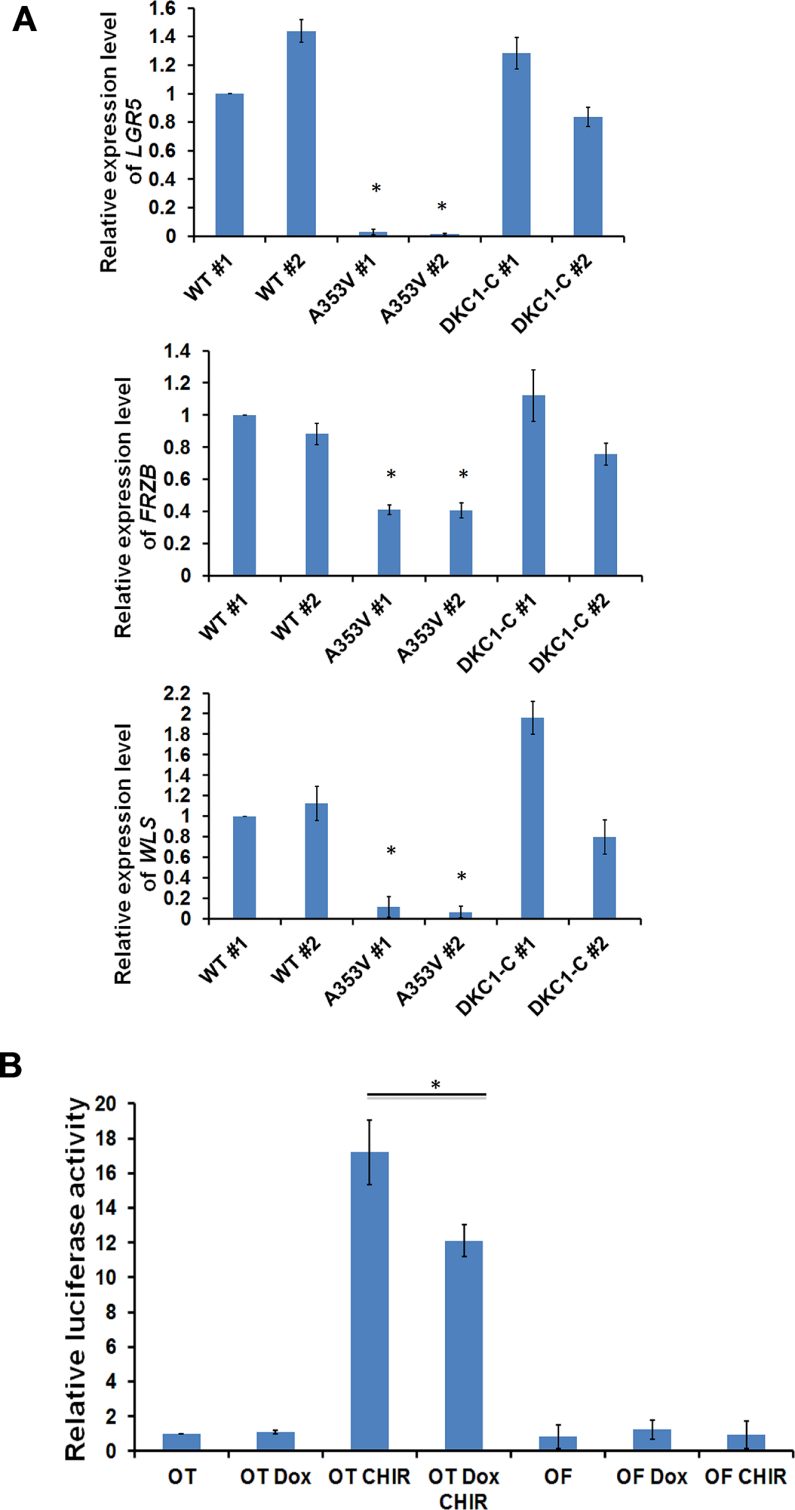
Expression of WT dyskerin can rescue the expression of *LGR5*, *WLS* and *FRZB*. A: Real-Time RT/PCR experiments showed that, in *A353V* iPS cells, the mRNA expression of *LGR5*, *FRZB* and *WLS* was significantly increased after expressing WT dyskerin protein. The combined results of 3 independent experiments are shown, the error bars show standard deviation. * p<0.01. B: Knock down of dyskerin affects canonical WNT signaling. The relative luciferase activity in inducible *DKC1*-shRNA HEK293T cells transfected with plasmids containing a luciferase gene under the control of a WT (OT-flash) or mutant (OF-flash) β-catenin responsive promotor and pRL-SV40 plasmid. Luciferase activity was measured by using dual-luciferase system according to protocol from manufacturer. OT: WT TCF/LEF reporter, OF: Mutant TCF/LEF reporter, Dox: Doxycycline, CHIR: CHIR-99021.Four independent experiments were carried out and the mean of results are shown; the error bars showing standard deviation. * p<0.01.

To gain insight into the pathways active in stem cells that are altered by mutant dyskerin we performed a high resolution microarray on the WT and mutant iPS cells using a GeneChip Human Transcriptome Array 2.0 (Affymetrix, USA). The original microarray data has been released to Gene Expression Omnibus (GEO) with accession number GSE66849 (http://www.ncbi.nlm.nih.gov/geo/query/acc.cgi?acc=GSE66849). Differentially expressed genes were analyzed by Ingenuity Pathway Analysis (IPA). The most striking difference was that several WNT signaling pathway genes, including *LGR5*, *FRZB*, *DKK1* and *WLS* were significantly decreased in all three *DKC1* mutant iPS cells compared to WT iPS cells (Table 1). WNT signaling plays a major role in development, cancer and stem cell renewal [36] and is therefore interesting from the point of view of DC pathogenesis. These results were validated by using real-time RT/PCR (Fig 4A). To study whether this altered WNT signaling is due to the dysfunctional telomerase or telomere, we tested iPS cells reprogrammed from DC patients with compound homozygous mutations of the *TERT* gene (*TERT CP*) which showed significantly decreased telomerase activity and shortened telomere length (S10 Fig). We found that the mRNA levels of these WNT related genes are significantly decreased; suggesting a dysfunctional telomerase complex can affect the WNT pathway (Fig 4A). Next, we wanted to exclude the possibility that this decreased expression of WNT related genes was caused by the consequence of the iPS cell reprogramming or differentiation. We made a HEK293T cell line in which a *DKC1* shRNA is induced by treatment with doxycycline and knocks down the dyskerin protein level by 80–90% as well as decreasing the telomerase activity (Fig 4B). After doxycycline treatment of two different *DKC1*-shRNA cell lines the mRNA levels of *LGR5*, *FRZB* and *WLS* were severely decreased while in *GFP*-shRNA control cells there was no difference. This result suggested that the decreased mRNA level of these genes is a direct consequence of the dysfunctional dyskerin protein. To further establish the relationship between mutant dyskerin protein and the lower level of WNT gene expression, we tested the mRNA level of these genes in our mutant iPS cells that were corrected by expressing a WT *DKC1* gene and found that, after expressing a WT *DKC1* gene in both *A353V* and *ΔL37* mutant iPS cells, the mRNA expression of *LGR5*, *FRZB* and *WLS* was fully restored (Fig 5A and S11 Fig). Interestingly the dominant negative effect of the *A353V* mutant, that we observed when measuring telomerase activity, was not evident. Finally, to study whether the decreased expression of WNT related genes caused by dysfunctional dyskerin could affect the WNT signaling pathway, we used a Top-flash luciferase reporter system [37] which responds to the canonical WNT signaling pathway. In our inducible *DKC1* shRNA 293T cells, we found that, although the relative luciferase activity did not show any difference after knocking down dyskerin, reduction of dyskerin levels can significantly block the response of the reporter system to GSK3b inhibitor, CHIR-99021, an aminopyrimidine derivative that is an extremely potent inhibitor of GSK3, inhibiting GSK3β and GSK3α and functions as a WNT pathway activator. (Fig 5B).

**Table 1:** Fold changes of WNT related genes in *DKC1* mutant iPS cells.

	Fold change in iPS cells with *DKC1* mutations compared to WT		
Gene	*A353V*	*ΔL37*	*Q31E*
*LGR5*	-3.12	-2.8	-2.96
*DKK1*	-2.08	-1.09	-1.33
*FRZB*	-1.97	-2.29	-1.31
*WLS*	-1.66	-1.26	-1.1
*PORCN*	-1.67	-1.59	-1.58
*RSPO3*	-1.56	-1.68	-1.3

## Discussion

In this paper we have investigated iPS cells reprogramed from fibroblasts of DC patients to see if they recapitulate features of the disease and hence if they will be a useful tool in investigating pathogenetic mechanisms and testing the efficacy of drugs. Several previous reports about iPS cells from DC patients have been published with some differences in the kinetics of telomere maintenance. Agarwal et al [38]found *DKC1* mutant iPS cells have elongated telomeres compared to fibroblast cells, but Winkler et al and Batista et al [39,40] reported telomere shortening and low *TERC* expression in *TERC*, *TERT* or *DKC1* mutated iPS cells, respectively. Here, we generated three different iPS cell lines from X-linked DC patient’s fibroblast cells carrying *DKC1 A353V*, *Q31E* and *ΔL37* mutations and showed that all three lines expressed low *TERC* RNA compared to WT controls. Telomere maintenance varied between the mutations; *Q31E* and *ΔL37* mutant iPS cells could not elongate telomeres as WT control cells do after reprograming and they maintain the same length as in the corresponding fibroblast. In *A353V* iPS cells, the telomere length is much shorter than in the corresponding fibroblast cells suggesting the *A353V* mutation more severely affects telomerase function. From our study and the others the consensus is that iPS cells recapitulate the effects on telomerase and telomere maintenance seen in DC and thus represent an abundant source of DC cells for further investigation. This is particularly valuable in the case of DC since other cells that can be obtained from patients, fibroblasts and transformed lymphocytes, do not express telomerase.

As well as telomere maintenance, dyskerin is essential for the maturation and modification of ribosomal RNA and there is a long-standing controversy concerning the importance of ribosome biogenesis and translation defects in the pathogenesis of DC. Mouse models with complete or partial knockdown of dyskerin[16,41], or those with mutations that mimic human pathogenic mutations[19,33] show delays in ribosome biogenesis and a decrease in the level of pseudouridine in mature rRNA. Reported downstream consequences of these defects are alterations in translation, notably decreased translation of mRNAs that use IRES elements for translation initiation[42,43]. Our previous work on mouse ES cells with *A353V*, *G402E* and *Δ15* mutations strongly suggested mutant dyskerin could cause significant ribosomal defects, including decreased pseudouridine levels in rRNA, delayed maturation of ribosome RNA, decreased expression of H/ACA snoRNA and altered rRNA migration pattern due to secondary structure changes[19,33,44]. Other investigators using cells from DC patients failed to show effects on ribosome biogenesis though these cells are scarce and difficult to obtain in reasonable quantities [20,21]. We reasoned that our iPS cells would be good cells in which to examine the effects of *DKC1* mutations on ribosome biogenesis. Surprisingly, we did not find a significant difference between WT and mutant iPS cells in the kinetics of rRNA processing or in pseudouridine levels in mature rRNA. We did observe slightly decreased expression of H/ACA snoRNA and scaRNA in mutant iPS cells. It is no surprise that severe knockdown of *DKC1* expression affects ribosome biogenesis but why do pathogenic mutations, when genocopied in mouse cells, affect ribosome biogenesis while the same mutations in humans do not? A likely explanation is that in humans only mutations that allow a certain level of ribosome production are viable. In this way mutations that preferentially affect telomere maintenance and leave ribosome biogenesis intact are selected, since they rarely affect embryonic development. The identical mutations in mouse dyskerin, which has 90% identity with the human protein, will likely have a more disruptive effect, if indeed human mutations have been selected as we suggest, and it is therefore no surprise they affect ribosome biogenesis and pseudouridylation, the major functions of dyskerin.

Of about 41 *DKC1* mutations that have been described [45] most are present in single, or a handful of, families but the *A353V* mutation is recurrent and accounts for 40 percent of all X-linked DC patients [5]. The phenotype of patients with the *A353V* mutation varies but tends to be severe, with some children showing the signs of classical DC and others showing the severe features of HH[5]. The lines of iPS cells with this mutation are therefore particularly promising if therapeutic approaches, such as gene correction, are pursued. Because *DKC1* is X-linked only one allele is expressed in either male or female cells so whether or not the mutations show a dominant negative effect has not been important. However when contemplating adding back the WT gene it is important to know if the mutations show a dominant negative effect. The *A353V* mutation clearly shows a dominant negative effect with respect to telomerase activity and telomere maintenance, likely because two molecules of dyskerin are present in each telomerase RNP particle. Interestingly no dominant negative effect was seen with respect to the decreased WNT signaling. Perhaps the partial recovery of telomerase is enough to restore WNT signaling.

The canonical WNT signaling pathway plays a prominent role in development, stem cell renewal and cancer [36]. In the absence of WNT signaling, β-catenin cannot accumulate in the cytoplasm since a destruction complex, including AXIN, APC, PP2A, GSK3b and CK1α degrades β-catenin via the ubiquitin/proteasome pathway. However, when WNT binds the frizzled receptor at the cell surface, the destruction complex function is disrupted and β-catenin accumulates in the nucleus and induces a cellular response through the TCF/LEF (T-cell factor/lymphoid enhancing factor) transcription factors. It has recently been shown that *TERT* transcription responds to WNT signaling in this way[46]. Our finding that *DKC1* mutations lead to decreased expression of frizzled receptors was unexpected. Perhaps telomerase levels stimulate transcription of frizzled receptors in proliferating cells to maintain high levels of WNT signaling and the telomerase needed for growth and proliferation via a self-maintaining cyclic mechanism. If this is disturbed by decreased telomerase levels then the transcription of receptor genes may decrease. It is interesting in this respect that the receptor most affected in the iPS cells is LGR5, a 7-transmembrane receptor. LGR5 has been implicated in the self-renewal of adult stem cells in the hair follicle, intestine and stomach[47,48] while the closely related LGR6 is active in stem cells of the sebaceous gland and skin[49]. Though neither protein has yet been associated with hematopoietic stem cells DC is associated with stem cell failure in other systems and has prominent skin, gut and hair manifestations.

We conclude that DC iPS cells represent a useful model of the DC stem cell and should accelerate research into the pathology of this devastating disease. Here we have shown that they recapitulate the telomerase defect in DC and show no defect in ribosome biogenesis. We find they show decreased expression of WNT receptors and WNT signaling which may be involved in the pathogenetic mechanism.

## Supporting Information

S1 Fig Telomere length of patients with *DKC1* ^A353V^ and *DKC1* ^Q31E^ mutations. Telomere lengths in PBMC were measured by flow cytometric fluorescence in situ hybridization. Telomere length in healthy control subjects between the ages of 1 day and 94 years. The 1st, 5th, 10th, 25th, 50th, 75th, 90th, 95th, 99th percentiles of healthy controls are shown.
(DOC)

S2 Fig *A353V*, *Q31E* and *ΔL37 DKC1* mutant iPS lines. A: Histological analysis of teratomas formed from mutant iPS cell lines, showing structures from all three germ layers. B: Pluripotency analysis of reprogrammed iPS cells. SSEA3 and SSEA4 antigens on the human pluripotent stem cell surfaces were examined by Flow cytometric assay.
(DOC)

S3 Fig Cytogenetic analysis of mutant iPS cells. Analysis of G-banded metaphase cells from mutant iPS clones at early passages showed normal karyotype. At least 20 metaphases of each iPS cell were examined.
(DOC)

S4 Fig Immunofluorescence staining of Dyskerin (red) and Fibrillarin (green) of iPS cells. DNA was counterstained with DAPI (blue).
(DOC)

S5 Fig Telomerase activity of Q31E iPS cells was measured by using the TRAP assay. IC: internal control. HI: Heat inactivated control. The quantitive data, derived by densitometry, are shown
(DOC)

S6 Fig Telomere length measurement of the Q31E iPS cells. Telomere length measurement of the Q31E iPS cells in different passages compared to those from the original fibroblast cells (Fib) by using pulse field gel electrophoresis and in-gel hybridization with telomere probe (TTAGGG)_3_.
(DOC)

S7 Fig SnoRNA Real time PCR. Real time RT/PCR results of some Cajal body snoRNA (U85, U90, U92 and U93) and C/D snoRNA (U16, snoRD124, U103b and U14) expression in WT and *DKC1* mutant iPS cells
(DOC)

S8 Fig Northern blot of 28S RNA of iPS cells. The RNA was extracted and mixed with RNA loading buffer and denatured at 65 degree for 3 minutes or 10 minutes, respectively, followed by separating on a 1.25% agarose gel and transferring to a nylon filter. An oligonucleotide complementary to 28S rRNA was used as a probe (5’-CACCTTTTCTGGGGTCTGAT-3’) in hybridization.
(DOC)

S9 Fig Nuclear localization of flag-tagged dyskerin. Flag tagged WT dyskerin located in the nucleolus of iPS cells after expression from the safe harbor AAVS1 site. Immunofluorescence staining of Flag (green) and Fibrillarin (red) of *A353V* and *ΔL37* iPS cells before and after expressing Flag-tagged Dyskerin. DNA was counterstained with DAPI (blue).
(DOC)

S10 Fig iPS cells with *TERT* ^R537H/2173-2187del15insACAG^ compound homozygotes mutation (*TERT CP*) showed decreased telomerase activity and shortened telomere length. A. Telomerase activity assay of WT and two *TERT-CP* iPS cells by using TRAP assay. 2×10^6^ cells were extracted by using CHAPS lysis buffer and serial diluted to indicate concentrations. IC: internal control, HI: heat inactivation,-: water control. B. Telomere length measurement of the iPS cells in different passages compared to those from the original fibroblast cells (F) by using pulse field gel electrophoresis and in-gel hybridization with telomere probe (TTAGGG)3.
(DOC)

S11 Fig Expression of WNT related mRNAs in *ΔL37* iPS cells. Real-time RT/PCR results showed that in *ΔL37* iPS cells, the mRNA expression of *LGR5*, *FRZB* and *WLS* was significantly increased after expressing WT dyskerin protein.
(DOC)
