# Supplementary material for: Impaired Telomere Maintenance and Decreased Canonical WNT Signaling but Normal Ribosome Biogenesis in Induced Pluripotent Stem Cells from X-Linked Dyskeratosis Congenita Patients
Source: PLoS One. 2015 May 18;10(5):e0127414. doi: 10.1371/journal.pone.0127414 (PMC4436374; doi:10.1371/journal.pone.0127414)
Supplement: S1 Fig — Telomere lengths in PBMC were measured by flow cytometric fluorescence in situ hybridization. Telomere length in healthy control subjects between the ages of 1 day and 94 years. The 1st, 5th, 10th, 25th, 50th, 75th, 90th, 95th, 99th percentiles of healthy controls are shown. (DOC) [file pone.0127414.s001.doc]

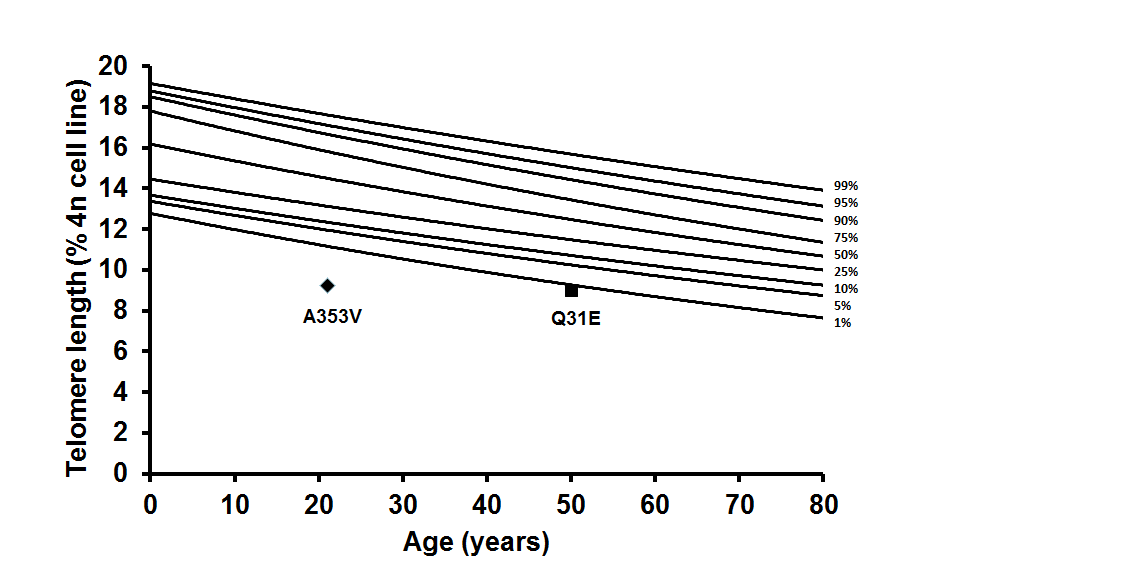


Supplementary Figure 1: Telomere length of patients with *DKC1^A353V^* and *DKC1^Q31E^* mutations. Telomere lengths in PBMC were measured by flow cytometric fluorescence in situ hybridization. Telomere length in healthy control subjects between the ages of 1 day and 94 years. The 1st, 5th, 10th, 25th, 50th, 75th, 90th, 95th, 99th percentiles of healthy controls are shown.
