# Supplementary material for: Impaired Telomere Maintenance and Decreased Canonical WNT Signaling but Normal Ribosome Biogenesis in Induced Pluripotent Stem Cells from X-Linked Dyskeratosis Congenita Patients
Source: PLoS One. 2015 May 18;10(5):e0127414. doi: 10.1371/journal.pone.0127414 (PMC4436374; doi:10.1371/journal.pone.0127414)
Supplement: S2 Fig — A: Histological analysis of teratomas formed from mutant iPS cell lines, showing structures from all three germ layers. B: Pluripotency analysis of reprogrammed iPS cells. SSEA3 and SSEA4 antigens on the human pluripotent stem cell surfaces were examined by Flow cytometric assay. (DOC) [file pone.0127414.s002.doc]

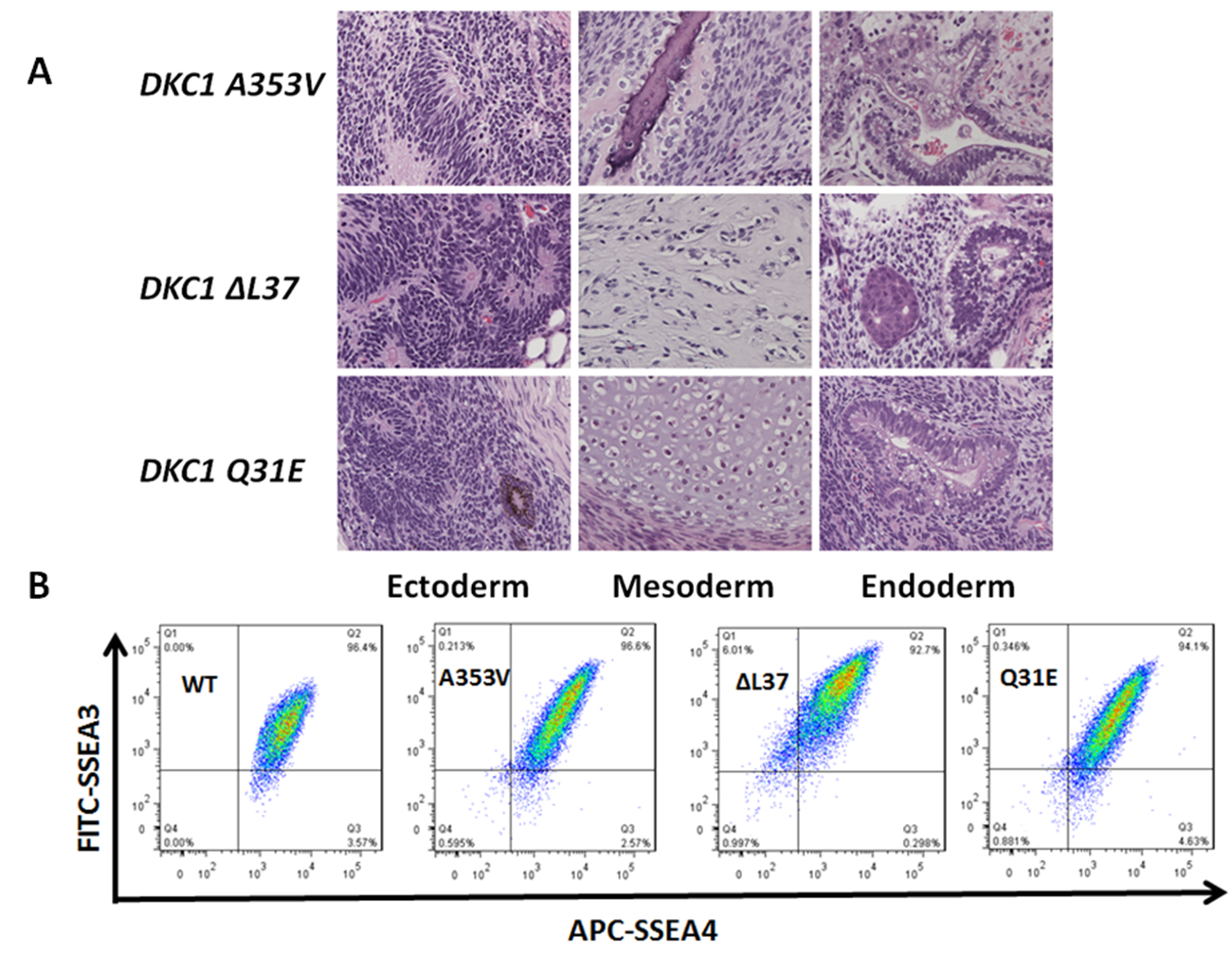


Supplementary Figure 2: *A353V*, *Q31E* and *ΔL37* *DKC1* mutant iPS lines. A: Histological analysis of teratomas formed from mutant iPS cell lines, showing structures from all three germ layers. B: Pluripotency analysis of reprogrammed iPS cells. SSEA3 and SSEA4 antigens on the human pluripotent stem cell surfaces were examined by Flow cytometric assay.
