# Supplementary material for: Impaired Telomere Maintenance and Decreased Canonical WNT Signaling but Normal Ribosome Biogenesis in Induced Pluripotent Stem Cells from X-Linked Dyskeratosis Congenita Patients
Source: PLoS One. 2015 May 18;10(5):e0127414. doi: 10.1371/journal.pone.0127414 (PMC4436374; doi:10.1371/journal.pone.0127414)
Supplement: S3 Fig — Analysis of G-banded metaphase cells from mutant iPS clones at early passages showed normal karyotype. At least 20 metaphases of each iPS cell were examined. (DOC) [file pone.0127414.s003.doc]

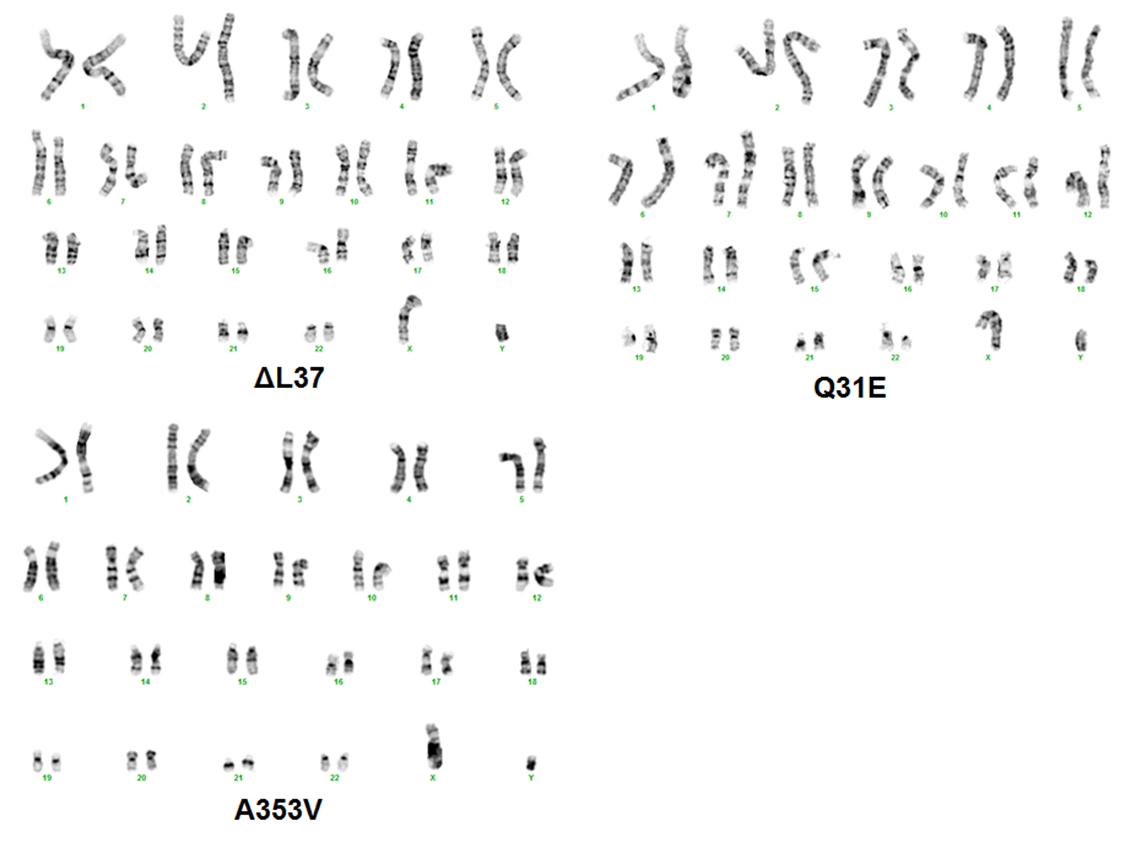


Supplementary Figure 3: Cytogenetic analysis of mutant iPS cells. Analysis of G-banded metaphase cells from mutant iPS clones at early passages showed normal karyotype. At least 20 metaphases of each iPS cell were examined.
