# Supplementary material for: Impaired Telomere Maintenance and Decreased Canonical WNT Signaling but Normal Ribosome Biogenesis in Induced Pluripotent Stem Cells from X-Linked Dyskeratosis Congenita Patients
Source: PLoS One. 2015 May 18;10(5):e0127414. doi: 10.1371/journal.pone.0127414 (PMC4436374; doi:10.1371/journal.pone.0127414)
Supplement: S4 Fig — DNA was counterstained with DAPI (blue). (DOC) [file pone.0127414.s004.doc]

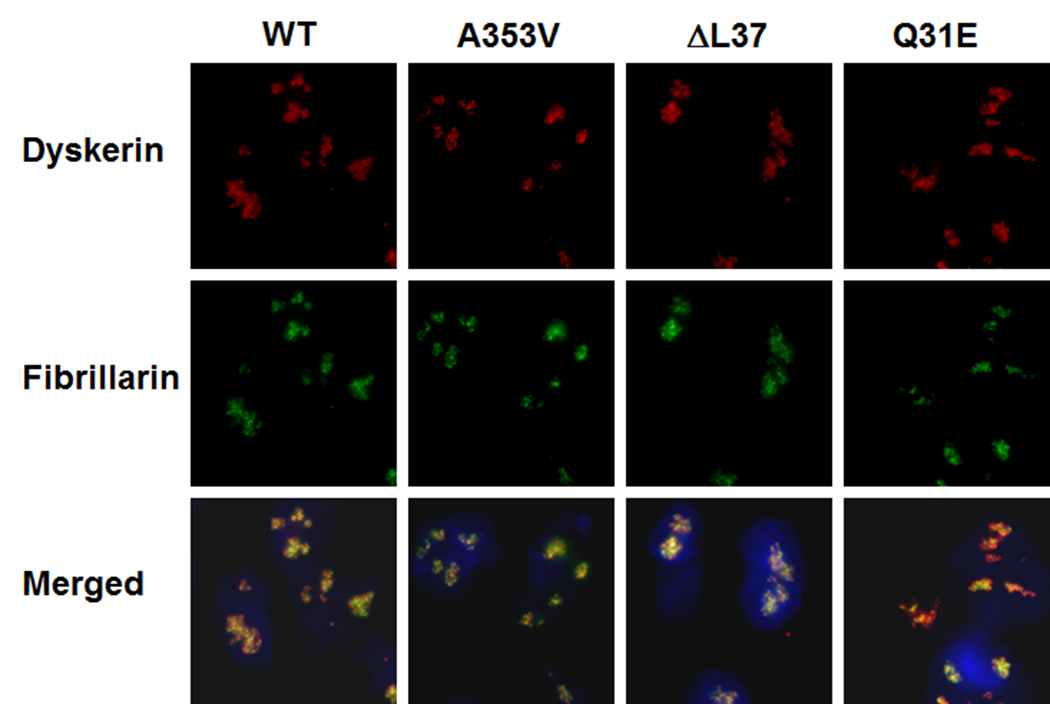


Supplementary Figure 4: Immunofluorescence staining of Dyskerin (red) and Fibrillarin (green) of iPS cells. DNA was counterstained with DAPI (blue).
