# Supplementary material for: Impaired Telomere Maintenance and Decreased Canonical WNT Signaling but Normal Ribosome Biogenesis in Induced Pluripotent Stem Cells from X-Linked Dyskeratosis Congenita Patients
Source: PLoS One. 2015 May 18;10(5):e0127414. doi: 10.1371/journal.pone.0127414 (PMC4436374; doi:10.1371/journal.pone.0127414)
Supplement: S5 Fig — IC: internal control. HI: Heat inactivated control. The quantitive data, derived by densitometry, are shown (DOC) [file pone.0127414.s005.doc]

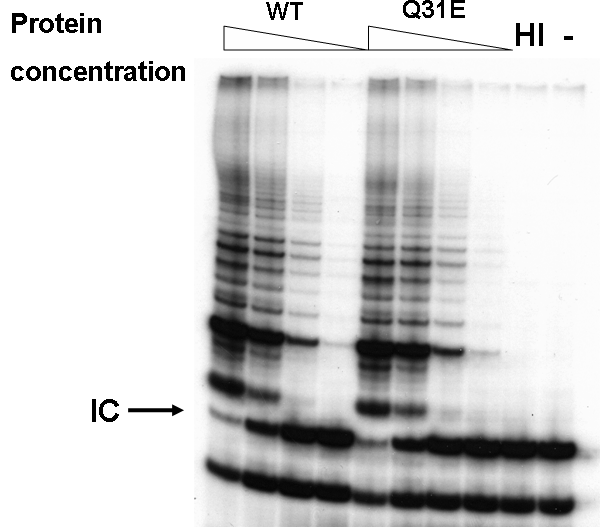


**1**

**0.98**

Supplementary Figure 5: Telomerase activity of Q31E iPS cells was measured by using the TRAP assay. IC: internal control. HI: Heat inactivated control. The quantitive data, derived by densitometry, are shown
