# Supplementary material for: Impaired Telomere Maintenance and Decreased Canonical WNT Signaling but Normal Ribosome Biogenesis in Induced Pluripotent Stem Cells from X-Linked Dyskeratosis Congenita Patients
Source: PLoS One. 2015 May 18;10(5):e0127414. doi: 10.1371/journal.pone.0127414 (PMC4436374; doi:10.1371/journal.pone.0127414)
Supplement: S6 Fig — Telomere length measurement of the Q31E iPS cells in different passages compared to those from the original fibroblast cells (Fib) by using pulse field gel electrophoresis and in-gel hybridization with telomere probe (TTAGGG)3. (DOC) [file pone.0127414.s006.doc]

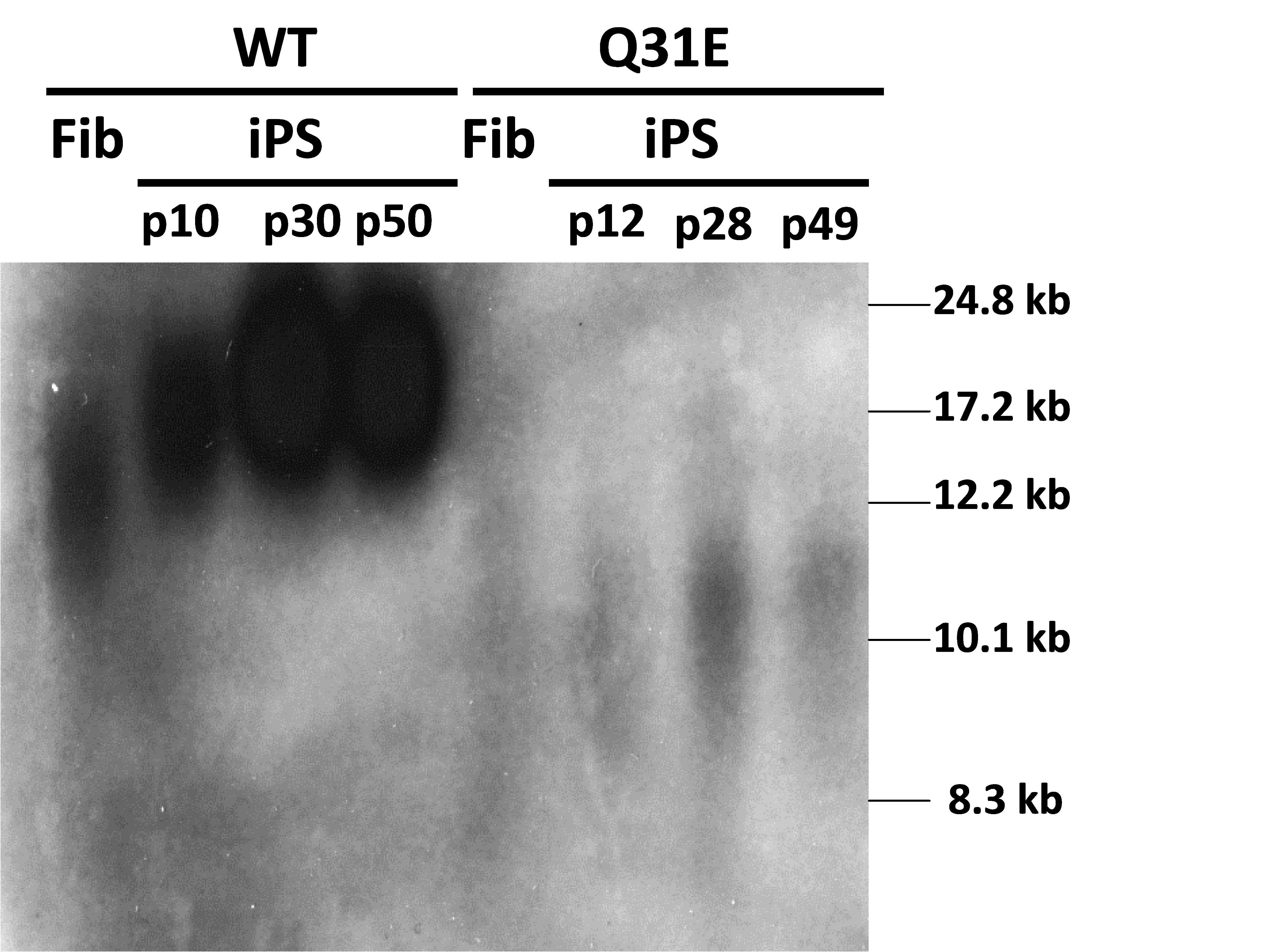


Supplementary Figure 6: Telomere length measurement of the Q31E iPS cells in different passages compared to those from the original fibroblast cells (Fib) by using pulse field gel electrophoresis and in-gel hybridization with telomere probe (TTAGGG)_3_.
