# Supplementary material for: Impaired Telomere Maintenance and Decreased Canonical WNT Signaling but Normal Ribosome Biogenesis in Induced Pluripotent Stem Cells from X-Linked Dyskeratosis Congenita Patients
Source: PLoS One. 2015 May 18;10(5):e0127414. doi: 10.1371/journal.pone.0127414 (PMC4436374; doi:10.1371/journal.pone.0127414)
Supplement: S7 Fig — Real time RT/PCR results of some Cajal body snoRNA (U85, U90, U92 and U93) and C/D snoRNA (U16, snoRD124, U103b and U14) expression in WT and DKC1 mutant iPS cells (DOC) [file pone.0127414.s007.doc]

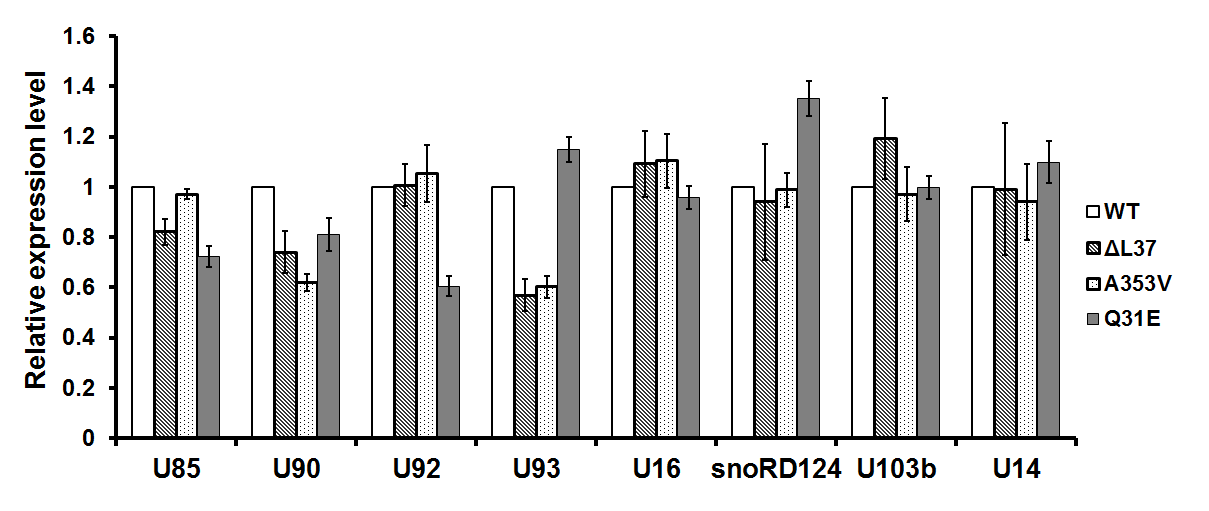


Supplementary Figure 7: Real time RT/PCR results of some Cajal body snoRNA ( U85, U90, U92 and U93) and C/D snoRNA ( U16, snoRD124, U103b and U14) expression in WT and *DKC1* mutant iPS cells
