# Supplementary material for: Impaired Telomere Maintenance and Decreased Canonical WNT Signaling but Normal Ribosome Biogenesis in Induced Pluripotent Stem Cells from X-Linked Dyskeratosis Congenita Patients
Source: PLoS One. 2015 May 18;10(5):e0127414. doi: 10.1371/journal.pone.0127414 (PMC4436374; doi:10.1371/journal.pone.0127414)
Supplement: S8 Fig — The RNA was extracted and mixed with RNA loading buffer and denatured at 65 degree for 3 minutes or 10 minutes, respectively, followed by separating on a 1.25% agarose gel and transferring to a nylon filter. An oligonucleotide complementary to 28S rRNA was used as a probe (5’-CACCTTTTCTGGGGTCTGAT-3’) in hybridization. (DOC) [file pone.0127414.s008.doc]

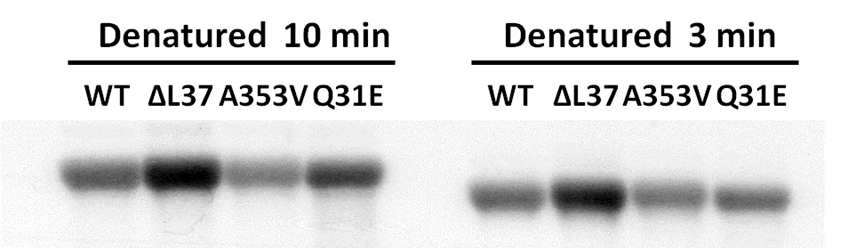


Supplementary Figure 8: Northern blot of 28S RNA of iPS cells. The RNA was extracted and mixed with RNA loading buffer and denatured at 65 degree for 3 minutes or 10 minutes, respectively, followed by separating on a 1.25% agarose gel and transferring to a nylon filter. An oligonucleotide complementary to 28S rRNA was used as a probe (5’-CACCTTTTCTGGGGTCTGAT-3’) in hybridization.
