# Supplementary material for: Impaired Telomere Maintenance and Decreased Canonical WNT Signaling but Normal Ribosome Biogenesis in Induced Pluripotent Stem Cells from X-Linked Dyskeratosis Congenita Patients
Source: PLoS One. 2015 May 18;10(5):e0127414. doi: 10.1371/journal.pone.0127414 (PMC4436374; doi:10.1371/journal.pone.0127414)
Supplement: S9 Fig — Flag tagged WT dyskerin located in the nucleolus of iPS cells after expression from the safe harbor AAVS1 site. Immunofluorescence staining of Flag (green) and Fibrillarin (red) of A353V and ΔL37 iPS cells before and after expressing Flag-tagged Dyskerin. DNA was counterstained with DAPI (blue). (DOC) [file pone.0127414.s009.doc]

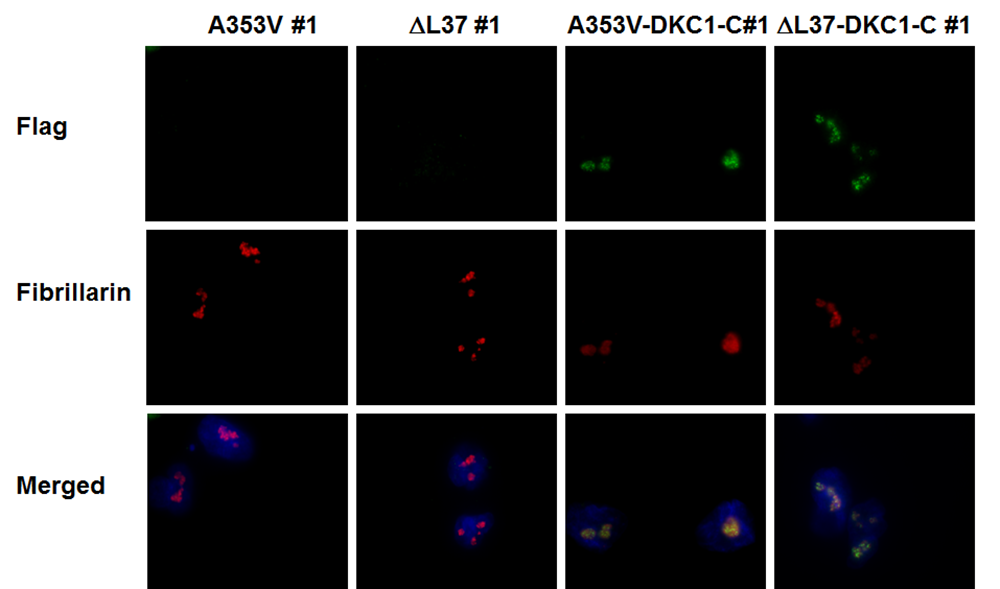


Supplementary Figure 9: Flag tagged WT dyskerin located in the nucleolus of iPS cells after expression from the safe harbor AAVS1 site. Immunofluorescence staining of Flag (green) and Fibrillarin (red) of *A353V* and *ΔL37* iPS cells before and after expressing Flag-tagged Dyskerin. DNA was counterstained with DAPI (blue).
