# Supplementary material for: Impaired Telomere Maintenance and Decreased Canonical WNT Signaling but Normal Ribosome Biogenesis in Induced Pluripotent Stem Cells from X-Linked Dyskeratosis Congenita Patients
Source: PLoS One. 2015 May 18;10(5):e0127414. doi: 10.1371/journal.pone.0127414 (PMC4436374; doi:10.1371/journal.pone.0127414)
Supplement: S10 Fig — A. Telomerase activity assay of WT and two TERT-CP iPS cells by using TRAP assay. 2×10^6^ cells were extracted by using CHAPS lysis buffer and serial diluted to indicate concentrations. IC: internal control, HI: heat inactivation,-: water control. B. Telomere length measurement of the iPS cells in different passages compared to those from the original fibroblast cells (F) by using pulse field gel electrophoresis and in-gel hybridization with telomere probe (TTAGGG)3. (DOC) [file pone.0127414.s010.doc]

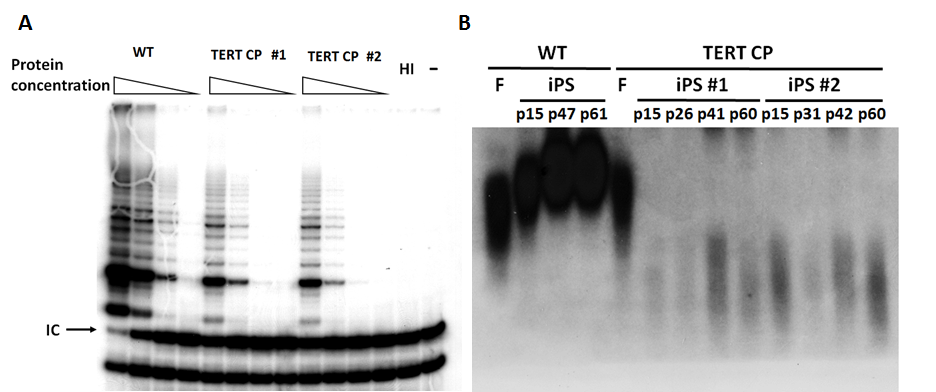


Supplementary Figure 10: iPS cells with *TERT^R537H/2173-2187del15insACAG^* compound homozygotes mutation (*TERT CP*) showed decreased telomerase activity and shortened telomere length. A. Telomerase activity assay of WT and two *TERT-CP* iPS cells by using TRAP assay. 2×10^6^ cells were extracted by using CHAPS lysis buffer and serial diluted to indicate concentrations. IC: internal control, HI: heat inactivation, -: water control. B. Telomere length measurement of the iPS cells in different passages compared to those from the original fibroblast cells (F) by using pulse field gel electrophoresis and in-gel hybridization with telomere probe (TTAGGG)3.
