# Supplementary material for: Impaired Telomere Maintenance and Decreased Canonical WNT Signaling but Normal Ribosome Biogenesis in Induced Pluripotent Stem Cells from X-Linked Dyskeratosis Congenita Patients
Source: PLoS One. 2015 May 18;10(5):e0127414. doi: 10.1371/journal.pone.0127414 (PMC4436374; doi:10.1371/journal.pone.0127414)
Supplement: S11 Fig — Real-time RT/PCR results showed that in ΔL37 iPS cells, the mRNA expression of LGR5, FRZB and WLS was significantly increased after expressing WT dyskerin protein. (DOC) [file pone.0127414.s011.doc]

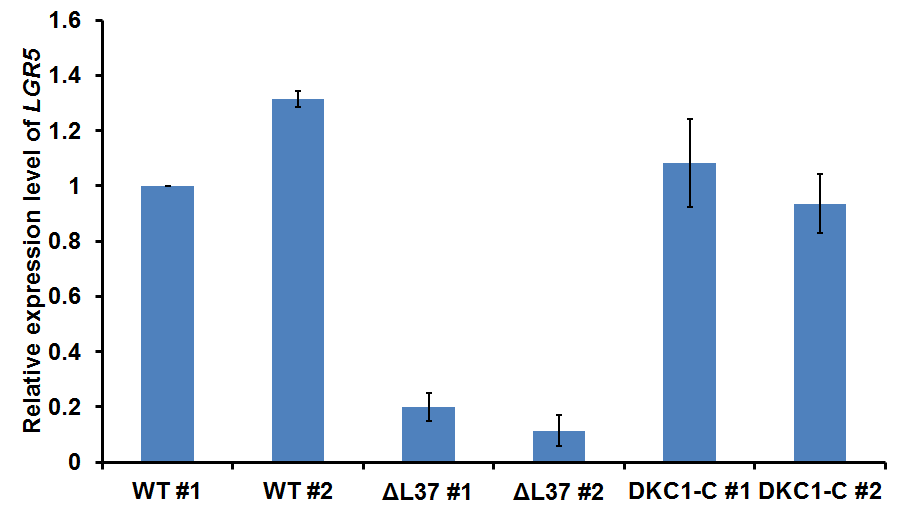


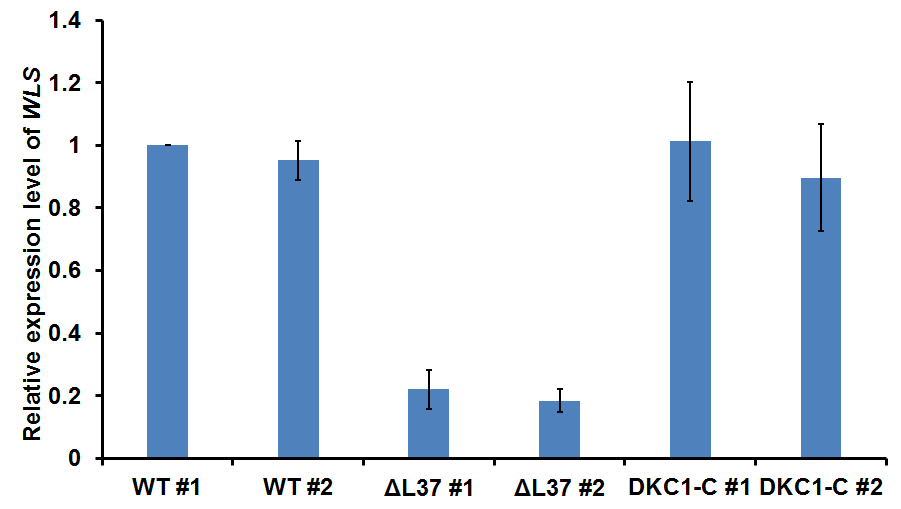


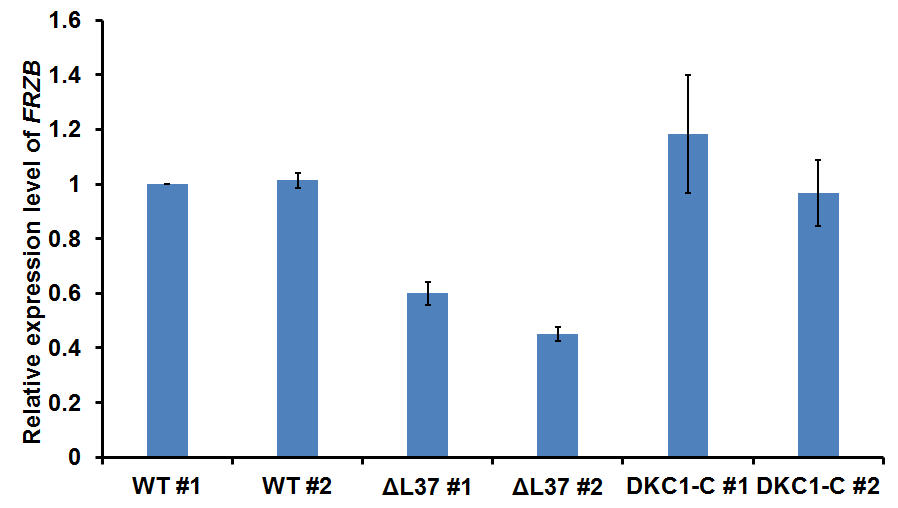


Supplementary Figure 11: Real-time RT/PCR results showed that in *ΔL37* iPS cells, the mRNA expression of *LGR5*, *FRZB* and *WLS* was significantly increased after expressing WT dyskerin protein.
